# The Place and Importance of Patients in Deliberative Processes

**DOI:** 10.3389/fmedt.2021.794695

**Published:** 2021-11-30

**Authors:** Marjo S. Cellier

**Affiliations:** Research Center of the Ste-Justine Hospital University Center Montreal, Montreal, QC, Canada

**Keywords:** patient partnership, patient and public involvement, ethics, deliberative process, HTA, decision making

## Introduction

“Perhaps the most notable wake-up call of all is inequity, as the worm in the heart of the world” (1). In the last year and a half, healthcare systems worldwide have been confronted to countless challenges and to the realization that their ways of making decisions are not always in line with the population's context, needs and priorities. As has been established in the past, patients being the main concerned stakeholders in the delivery of their care, not only is it their right to be included in deliberative processes that will impact them (2), their implication and the insight they provide are the key to ethical decision making, which is the only sustainable solution to inequities in healthcare. However, patient involvement is not sufficient to ensure accurate and long-lasting representativity of the population's needs and priorities. As was identified in 2020 (3), the “Need to design better approaches to involve stakeholders in HTA” is a major challenge of health technology assessment (HTA). INAHTA recently released a position statement on the subject, and recognized patient involvement as an “important and valuable element in the conduct of HTA,” providing a list of important considerations for meaningful patient involvement (4). In addition, a collective reflection on legitimacy, values and patient involvement in HTA, including in deliberation for clinical practice guidelines (CPG), was proposed to tackle ethical challenges and develop deliberative processes focused on patients and population needs (5). If a decision is to be fair and reasonable, as defined by the ethical framework accountability for reasonableness (A4R) (6), a consistent and long-term partnership with patients, implying collaboration, communication and ensuring patients are listened to, must be applied throughout every step of the deliberative process.

Any partnership starts with a respectful relationship and implies equal collaboration. If decisions in healthcare are made based on anything other than an accurate representation of the contexts, priorities and needs of the impacted population, when translated into policy, there will be oversights of potentially essential principles, and therefore failures in providing the best care for the largest population possible. Oversights of certain specifics which make care acceptable and efficient for all are understandably made when patients are not involved, or if their involvement is limited, because no matter how good the intentions, experts in deliberative processes lack outside view of the results of decisions that are made, and of the receiving end of their translation into policy and care.

## Deliberative Processes

Because of the complexity of deliberation processes, patients are rarely involved beyond a consultation role, which is indeed essential, but is limited in its impact and cannot ensure sufficient representativity of the needs of the population to determine the best possible provision of care. The experiential knowledge patients can provide in the form of data can shape deliberative processes and provide a strong base for understanding their context and for prioritization of their needs. However, the accuracy and value of this data is determined by its representativity of the actual population, which requires an adequate diversification process to ensure all voices are heard. Every sub-group, determined by diversification criteria applied to a general population, must be able to provide their opinions and insights in an environment conducive to debate and discussion on what they need and what the deliberative process should prioritize in further steps.

An ethical framework designed to assess how an intervention contributes to the foundational objective of healthcare systems through several dimensions (7) provides a single structured method to be used for every step of a deliberative process. Such structure is essential to ensure continuity and coherence, to provide a tool for collection, analysis, and synthesis of data, as well as a basis for discussion through deliberation. The analysis of data is the core of deliberative processes and must therefore be able to identify the essential aspects and priorities to be considered in the making of decisions. Data collected throughout the initial steps of deliberative processes must then be synthesized in order to see emerge the essential points and considerations to be taken into account for accurate decision making, and to ensure the result is applicable in the context of the population and in line with its current needs.

To allow key aspects to emerge, they must be organized in a way that can reveal where gaps are in the current context of the population, and where inconsistencies or issues in the delivery of care must be addressed. The essential points and considerations identified through the analysis can therefore be prioritized to facilitate the decision making process. The insight and the opinions of patients throughout this process are essential to ensure that the prioritization of these key arguments is representative of what their needs and priorities are. However, if patients are consulted to provide data that will only be used to fill potential gaps in the research, or as a way to justify decisions that, in the end, aren't in line with what they ask for or what they need, there's no point. Structuring the data in a framework prevents this from happening, and will allow for conducive and consistent partnership with patients throughout each step of the deliberative process if the tools for the collection of data, for its analysis and for the synthesis, are constructed following the same structure and are organized in linear steps, in order to follow a logical train of thought which can ensure nothing gets lost in translation.

A multi-dimensional method following this principle provides a way to reflect on every aspect of healthcare provision, such as the current socio-political context, the populational contexts, needs and priorities, clinical benefits, constraints and acceptability, as well as organizational and economic requirements. Experiential data and insights provided by patients impact on every one of these dimensions and help avoid any oversight of seemingly small or insignificant detail that could impact their care or its provision, as well as ensure no erroneous assumptions will be made regarding the needs of the target population. These five dimensions, Socio-political, Populational, Clinical, Organizational and Economic, put together, are the pillars upon which rest fair and reasonable decisions (7) when they are used for careful consideration of every aspect of care as identified by the patients, of their potential or reported impacts, and of all necessary requirements for sustainable and acceptable provision of care within the healthcare system. The acceptability of care is central to making informed decisions regarding its provision since it's defined by individual experience of care and its impacts. However, experts in deliberative processes alone cannot ensure that patients' needs and priorities are at the center of the entire process, especially when HTA processes have had a tendency to focus on quantitative scientific literature to sustain their conclusions, as has been the traditional way for most of HTA natural history (8).

A method which allows all types of data to be analyzed in the same way creates an ensemble view of the subject and of all its implications. Looking at every aspect of provision of care is paramount to make decisions which will be in line with the population and their needs. This is why patients must be involved in the process of data analysis; to provide insight for every one of these aspects, to validate the prioritization of key points emerging from the data, and to ensure the needs of the population are kept at the top of the list of prioritized aspects to be considered and discussed in final decision making processes.

The deliberative process' final step is the deliberation itself, where aspects which have been qualified as essential through analysis and synthesis of the data are discussed, and conclusions are made in order to make informed decisions. When patients are involved in the deliberation discussions, as a way to validate the interpretations of conclusions made from prioritization of key aspects through the analysis and synthesis, they can maintain the population's needs at the center of the discussions, and as resulting basis for decision making, which can avoid making decisions that could have been made based on biased views of the quality or importance of different types of data. Such decisions, which are “evidence-informed” (9), but not patient centered can result in translation into policy and care that would not answer all the needs, communicated by the patients, of the target population, which is neither fair nor reasonable. An ethical decision in healthcare can only be made through careful consideration of the needs and priorities of the population it impacts.

The key issue is that patients are seldom solicited to participate in reflection on every dimension of a subject (sociocultural, populational, clinical, organizational, economic) during a deliberation, and thus are not empowered to contribute to the balancing act that leads to fair and reasonable decisions. A recent example demonstrated that it is possible to do so by cultivating a relationship with key patients, representative the entire population, throughout every step of the deliberative process, starting from engaging into meaningful discussion during the consultation and data collection process, up to the deliberation, hence giving them the opportunity to stand their grounds in the presence of healthcare professionals and/or experts included around the deliberative table. With attentive and respectful listening to patients' experiential knowledge and viewpoints, assumptions regarding what is best or preferred by patients made without their direct perspective no longer have their place in the discussion, and the legitimacy, fairness and usefulness of the decisions are enhanced (10).

## Discussion

It can often be believed that decisions in healthcare are unfortunately not always solely based on patient best interest and welfare (6), which is a grave deviation from the very purpose of our healthcare systems, and needs to be remedied. Every decision made in healthcare that translates into policy and care impacts patients, and they must be present at every step of the deliberative processes leading up to decision making, since their presence can lead to avoiding countless opportunities for oversights, assumptions or misinterpretations which can have dramatic repercussions if not caught. The objective of HTA has always been to try and determine the best ways to provide care to patients, which has often been articulated as the Triple Aim: Care; improving the individual experience of care, Health; improving the health of populations and Cost; reducing the cost of care for populations (11). The best way to satisfy these conditions is quite simple when considering its basic principle: providing the best care, for the most people, spending the least money. Improving the individual experience of care for patients is something only they can determine how to do, and which can be implemented through considering their perspectives and advice on how to best satisfy the different needs of the population when developing the specific aspects of provision of care. Improving the health of the population requires accessible and acceptable care for all concerned patients, which in turn depends on developing the best possible individual experience of care for the entire population. Finally, reducing cost of care implies sustainability on the long-term of the most acceptable and efficient version of care, which is determined by the optimal individual experience of care. The careful consideration of insights and experiential knowledge provided by patients on their own care, based on their needs and priorities, throughout deliberative processes and final decision making regarding development and provision of care is a step toward ensuring these aims being met. HTA is a process which, by definition, can always be improved (12).

Although it is methodology experts who develop, apply and improve methods used in these processes, the patients are the ones who live with the decisions that are made, and the ones best suited to determine whether they will improve healthcare (13). Developing a solid and, most importantly, equal partnership of collaboration and respect with patients throughout these processes is key to making truly representative and therefore ethical decisions for everyone.

## Author Contributions

The author confirms being the sole contributor of this work and has approved it for publication.

## Conflict of Interest

The author declares that the research was conducted in the absence of any commercial or financial relationships that could be construed as a potential conflict of interest.

## Publisher's Note

All claims expressed in this article are solely those of the authors and do not necessarily represent those of their affiliated organizations, or those of the publisher, the editors and the reviewers. Any product that may be evaluated in this article, or claim that may be made by its manufacturer, is not guaranteed or endorsed by the publisher.
